# Predictors and Outcomes of Invasive Mechanical Ventilation in Opioid Overdose Hospitalization in the United States

**DOI:** 10.7759/cureus.9788

**Published:** 2020-08-16

**Authors:** Adeolu O Oladunjoye, Olubunmi O Oladunjoye, Oluwatoyin Olubiyi, Maria Ruiza Yee, Eduardo D Espiridion

**Affiliations:** 1 Medical Critical Care, Boston Children's Hospital, Harvard Medical School, Boston, USA; 2 Psychiatry, Reading Health Tower Health, West Reading, USA; 3 Internal Medicine, Reading Hospital Tower Health, West Reading, USA; 4 Public Health, Philadelphia Department of Health, Philadelphia, USA; 5 Psychiatry, Philadelphia Collge of Osteopathic Medicine, Philadelphia, USA; 6 Psychiatry, Reading Hospital Tower Health, West Reading, USA; 7 Psychiatry, Drexel University College of Medicine, Philadelphia, USA; 8 Psychiatry, West Virginia School of Osteopathic Medicine, Lewisburg, USA; 9 Psychiatry, West Virginia University School of Medicine, Martinsburg, USA; 10 Psychiatry, Philadelphia College of Osteopathic Medicine, Philadelphia, USA

**Keywords:** hospitalization, opioid, opioid overdose, invasive mechanical ventilation

## Abstract

Introduction

Opioid overdose is increasingly becoming common and so is the need for invasive mechanical ventilation (IMV) for opioid overdose admissions in hospitalized patients. Respiratory failure requiring invasive mechanical ventilation is the most common reason for the admission of opioid-associated overdose patients. The aim of our study was to assess the demographic and clinical characteristics associated with the increased need for IMV in hospitalized opioid overdose patients.

Methods

We analyzed all adult admissions (18 years and above) using the National Inpatient Sample (NIS) database for five years from January 1, 2010-December 31, 2014 to identify opioid overdose patients requiring invasive mechanical ventilation. We compared the demographic and clinical characteristics of opioid overdose patients requiring and not requiring mechanical ventilator support and performed univariate and multivariate analyses to determine the odds ratio (OR) of association.

Results

A total of 2,528,751 opioid overdose patients were identified among which 6.4% required IMV during hospitalization. The prevalence of opioid overdose and the need for IMV increased by 31% and 38%, respectively, over the study period. Multivariate logistic regression (OR (95% CI), *p*<0.001) determined the following to be associated with increased odds of mechanical ventilator use: (OR 1.12 (1.06-1.19)) among patients aged 25-39 years vs (1.36 (1.28-1.44)) for the age group 40-64 years when compared to 18-24 years; hospital locations in the south US region (OR 1.62 (1.49-1.75)) when compared to the northeast US region; the presence of aspiration pneumonia (OR 14.30 (13.63-15.0)), rhabdomyolysis (3.22 (3.04-3.42)), septic shock (9.15 (8.41-9.97)), and anoxic brain injury (15.5 (13.70-17.50)). Other factors associated with decreased odds of IMV include hepatitis C virus infection (OR 0.75 (0.72-0.79)) and black race (OR 0.68 (0.63-0.74)]. Opioid overdose patients requiring IMV had a higher length of stay by 8.9 ± 0.1 days, higher hospitalization cost by US$ 28,117.81 ± 373.53, and higher in-hospital mortality rate (13.4% vs 0.3%).

Conclusion

The prevalence of opioid overdose and the need for IMV increased over the five-year study period, reflecting an increase in the relatively high in-hospital mortality of opioid overdose patients on IMV. Patient’s age, geographic location, race, and several comorbidities affect the need for invasive mechanical ventilation in hospitalized opioid overdose patients. These findings emphasize the need for a better understanding of these risk factors in creating a strategic approach for hospital care of opioid overdose patients.

## Introduction

Drug overdose is the leading cause of injury-related fatality in the United States (US) after a motor vehicle accident [1]. In 2018, 70% of US drug overdose deaths involved opioid use [2]. Opioid overdose has been on the rise since the 1990s, leading to different waves of opioid-associated overdose deaths. Over a decade since the 2000s, the number of deaths has quadrupled [3]. The US health system has taken a hit with more than 9 Billion dollars spent as of 2005 on the epidemic [3]. The second wave of the epidemic experienced a rapid rise in opioid overdose deaths, starting in 2010 with the increased use of heroin [4]. Unfortunately, the southwestern United States and Appalachia saw the largest per capita increase in the death rate due to drug overdose [5]. This second wave continued to rise and then the third wave took over in 2013 due to prescription or illicitly manufactured synthetic opioids, tramadol, and fentanyl made available to people presenting with pain in the healthcare facility [6].

While national attention is focused on the treatment given by first responders, not enough national attention is given to those who might need hospitalization to treat complications from an opioid overdose. The need for invasive mechanical ventilation (IMV) for opioid overdose admissions is increasing, as the opioid cases on hospitalization also increase. This has driven the overall cost of caring for these patients who need critical intensive care from about an average of $58,000 in 2009 to $92,400 in 2015 [7]. Respiratory failure requiring invasive mechanical ventilation is the most common reason for admissions of opioid-associated overdose [3]. This requirement has led to major causes of morbidity and mortality in hospitalized patients presenting with opioid overdose [1].

However, little is known about the predictors and outcomes of hospitalized patients who need mechanical ventilation during opioid overdose hospitalization. Therefore, we sought to assess the predictors and outcomes associated with the use of invasive mechanical ventilation in opioid overdose patients hospitalized in the United States.

## Materials and methods

Study design and data sources

We analyzed all adult admissions (18 years and above) using the Healthcare Cost and Utilization Project - National Inpatient Sample (HCUP-NIS) database from January 1, 2010, to December 31, 2014. This database is sponsored by the Agency for Healthcare Research and Quality (AHRQ). It is the largest all-payer publicly available inpatient care database made up of a 20% sample of US hospitalizations, from more than 40 (of the 50) states in the US providing a weighted estimate representing >95% of the hospitalized population.

The International Classification of Diseases Ninth Revision Clinical Modification (ICD-9-CM) was derived from 15 procedure columns and 25-30 diagnoses columns, which were used to identify the study population. Since the database is de-identified and publicly available, ethical clearance or Institutional Review Board approval was not necessary.

Study population and characterization of variables

**Figure 1:**
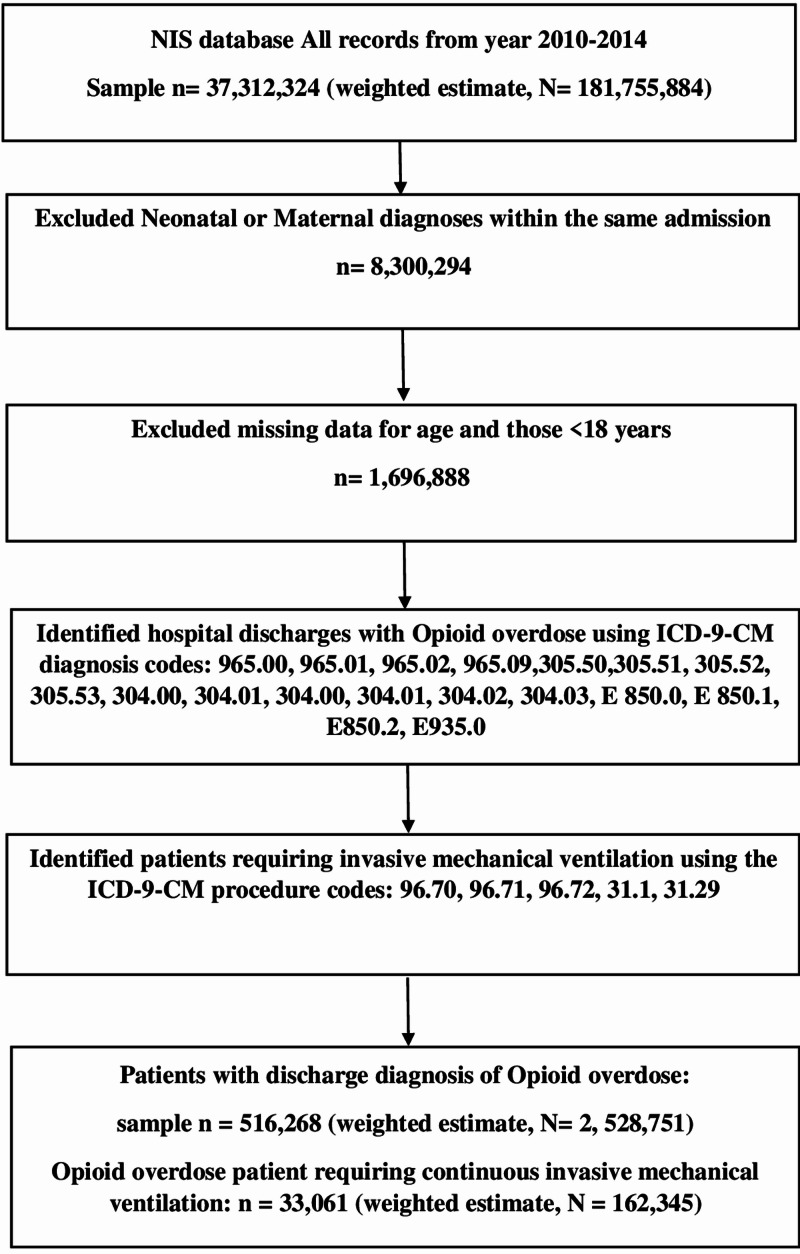
Flowchart for invasive mechanical ventilation in opioid overdose hospitalizations in the United States

The following International Classification of Diseases Ninth Revision Clinical Modification (ICD-9-CM) diagnosis codes were used to identify the diagnosis for opioid overdose patients: presence on admission of prescription overdose (ICD-9 965.00, 965.09, or E code E 850.0, E 850.1, and E850.2); heroin overdose (ICD-9 965.01, E 850.0, and E 935.0); methadone overdose (965.02); non-dependent opioid abuse (ICD-9 305.50,305.51, 305.52, and 305.53); and opioid type dependence (ICD-9 304.00, 304.01, 304.00, 304.01, 304.02, and 304.03) as used in previous literatures (Figure 1) [7-8]. We also identified procedure codes that enabled us to derive those who used invasive mechanical ventilation. These include 96.70, 96.71, 96.72, 31.1 and 31.29.

Our primary outcomes of interest were assessing the demographic and clinical characteristics associated with the increased need for IMV in hospitalized opioid overdose patients. Our secondary outcomes of interest were the incidence of in-hospital mortality and the discharge disposition of hospitalized opioid overdose patients who needed IMV. We had two groups for comparison among the opioid overdose patients based on whether they used IMV or not during their hospitalization. Demographic and clinical characteristics were then compared to assess the difference between these two groups, with univariate and multivariate analyses reported to show their differences and associated statistical significance (OR 95%CI; P = 0.005).

We also sort to report the prevalence and hospitalization trends among opioid overdose patients and show trends for those who had IMV during their hospitalization. Tables and figures were generated to show our results.

Patient demographics and comorbidities

Patients demographics include age (divided into 18-24, 25-39, 40-64 and 65+ years), race (white, black, and others), insurance provider (divided into government, private, self-pay, and others), income (divided into four quartiles), regions of the US (northeast, south, midwest/north-central, and west). Clinical characteristics were derived from the database for patients who had hepatitis C virus infection (HCV) (ICD-9 070.41, 070.44, 070.51, 070.54, 070.70, and 070.71) and complications of opioid overdose including aspiration pneumonia (ICD-9 507.0), septic shock (ICD-9 785.52), rhabdomyolysis (ICD-9 728.88), and anoxic brain injury (ICD-9 348.1 and 437.9).

Statistical analysis

All statistical analyses were performed using Stata version 15.0 (StataCorp, College Station, TX). We reported the mean and standard deviation for continuous variables. We used a P-value of <0.05 and a 95% confidence interval (CI). Statistical tests, such as the chi-square test and percentages, were used to report categorical variables. The student’s t-test was used for numeric variables. Linear models were used to derive trend analysis using the Joinpoint regression analysis statistical software (Belleair Beach, Florida) to derive the annual percentage change (APC). The APC considers constant changes that occur over a specified period in which the rate of disease change is seen in relation to the years assessed in percentages. Joinpoint software takes trend data and, based on the maximum number of joinpoints supplied by the user, fits the data into segments, enabling the users to assess if the apparent change in trend is statistically significant [9]. All analyses were performed with strata and weight to account for the complex clustered sampling methodology.

## Results

We studied a total of 2,528,751 opioid overdose patients identified for the five-year study period from January 1, 2010, to December 31, 2014, among which 162,345 (6.4%) required IMV during hospitalization. There were 54.7% male, 69.6% white, and 52.5% of the total population aged 40-64 years in these hospitalized patients. The mean age of overall patients is 44.6±0.1 years. The demographic and clinical characteristics were compared between those who were and were not on IMV during their hospitalization for opioid overdose (Table 1).

**Table 1:** Baseline characteristics of hospitalized opioid-associated overdose patients stratified by use of invasive mechanical ventilation (IMV) n, sample number; N, weighted average; HCV, hepatitis C virus

Name	Overall (n= 516,268) (N= 2, 528,751)	IMV (n= 33,061) (N= 162,345)	No IMV (n=483,207) (N=2,366,406)		P-Value
Mean Age (±SE), y	44.6±0.1	46.3±0.1	44.4±0.1		<0.0001
Age, y					<0.0001
18-24	9.2	7.6	9.3		
25-39	29.7	26.0	30.0		
40-64	52.5	56.9	52.3		
≥ 65	8.5	9.4	8.5		<0.0001
Sex, %					
Male	54.7	55.3	54.7		
Female	45.3	44.7	45.3		0.1499
Race, %					
White	69.6	75.5	69.2		
Black	17.1	11.7	17.4		
Other	13.3	12.8	13.4		<0.0001
Comorbidities, %					
HCV	16.7	15.1	16.9		<0.0001
Complication					
Aspiration Pneumonia	3.7	28.9	1.9		<0.0001
Septic Shock	1.2	11.1	0.5		<0.0001
Rhabdomyolysis	2.9	13.9	2.2		<0.0001
Anoxic Brain Injury	0.8	9.8	0.2		<0.0001
Income, %					
First quartile	35.0	35.3	35.0		
Second Quartile	24.9	26.1	24.8		
Third Quartile	23.4	22.6	22.3		
Fourth Quartile	17.8	16.0	17.9		<0.0001
Insurance, %					
Government	60.7	60.0	60.7		
Private	19.4	18.8	19.5		
Self-Pay	13.6	15.4	13.5		
Others	6.3	5.8	6.3		<0.0001
Region, %					
North East	27.2	19.2	27.7		
Mid-West/North Central	21.2	21.0	21.2		
South	31.2	36.4	30.9		
West	20.4	23.4	20.2		<0.0001
Hospital Teaching Status, %					
Rural	7.9	8.1	7.9		
Urban Non-Teaching	36.4	35.7	36.5		
Urban Teaching	55.7	56.2	55.6		0.5961
Discharge Disposition, %					
Home/Home Health	83.6	62.3	84.9		
Others	16.4	37.7	15.1		<0.0001
Length of Stay, Days	5.3±0.1	8.9±0.1	5.1±0.0		<0.0001
Cost (± SE)	9673.67±138.70	28117.81±373.53	8412.97±122.32		<0.0001
Mortality, %	1.2	13.4	0.3		<0.0001

Patients with opioid overdose on IMV during hospitalization

**Figure 2:**
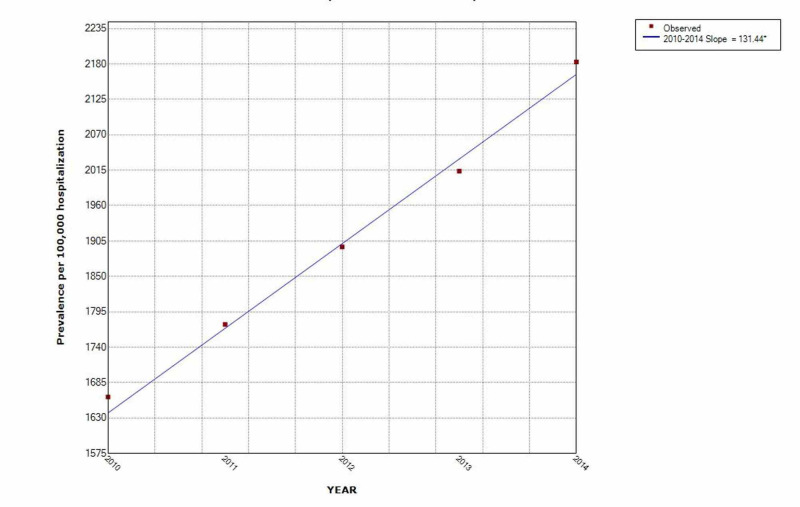
Increasing trend of opioid overdose hospitalization over the five-year study period * indicates that the slope is significantly different from zero at the alpha = 0.05 level. Final selected model: 0 Joinpoints

**Figure 3:**
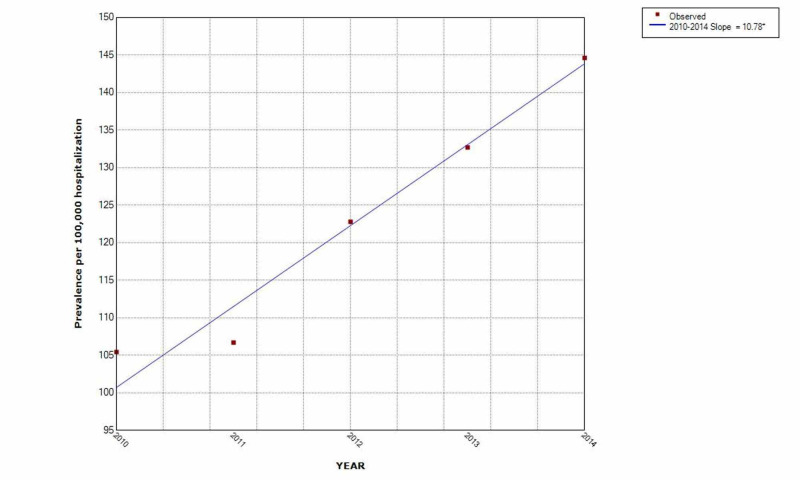
Increasing trend of opioid overdose hospitalization in those on IMV use over the five-year study period * indicates that the slope is significantly different from zero at the alpha = 0.05 level. Final selected model: 0 Joinpoints IMV: invasive mechanical ventilation

The prevalence of IMV use in opioid overdose increased year after year. It increased by 38% and the prevalence of opioid overdose increased by 31% over the five-year study period. (Figures 2-3)

After adjusting for other variables, multivariate logistic regression (OR (95% CI), p<0.001) determined the following to be associated with increased odds of mechanical ventilator use: age group 25-39 years (OR 1.12 (1.06-1.19)) and 40-64 years (OR 1.36 (1.28-1.44)) as compared to 18-24 years; hospitals in the US south region (OR 1.62 (1.49-1.75)), US mid-west/north-central region (OR 1.57 (1.42-1.74)), and US west region (OR 1.38 (1.27-1.50)) as compared to the northeast region. However, there was no statistically significant difference in income, insurance, or hospital teaching status.

Patients hospitalized for opioid overdose had comorbidities, including 3.7% having aspiration pneumonia, 2.9% had rhabdomyolysis, 1.2% had septic shock, and 0.8% had an anoxic brain injury. Also after adjusting for other variables, these comorbidities were associated with increased odds of mechanical ventilator use: aspiration pneumonia (OR 14.30 (13.63-15.0)), rhabdomyolysis (OR 3.22 (3.04-3.42)), septic shock (OR 9.15 (8.41-9.97)), anoxic brain injury (OR 15.5 (13.70-17.50)). However, on the other hand, those who had hepatitis C virus infection (OR 0.75 (0.72-0.79)) and black patients (OR 0.68 (0.63-0.74), had reduced odds of invasive ventilation (p<0.001) (Table 2).

**Table 2:** Factors associated with invasive mechanical ventilators in hospitalized opioid-associated overdose patients HCV, hepatitis C virus; HBV, hepatitis B virus

Name	Univariate analysis (Crude OR)	P-Value	Multivariate analysis (Adjusted OR)	P-Value
Mean Age (±SE), y	1.01 (1.01-1.01)	<0.0001		
Age, y				
18-24	Ref			
25-39	1.08 (1.01-1.13)	0.018	1.12 (1.06-1.19)	<0.0001
40-64	1.34 (1.26-1.42)	<0.0001	1.36 (1.28-1.44)	<0.0001
≥ 65	1.37 (1.28-1.47)	<0.0001	1.01 (0.93-1.09)	0.7930
Sex, %				
Male	Ref			
Female	0.98 (0.95-1.01)	0.1510		
Race, %				
White	Ref			
Black	0.61 (0.56-0.67)	<0.0001	0.68 (0.63-0.74)	<0.0001
Other	0.88 (0.83-0.93)	<0.0001	0.94 (0.89-1.00)	0.0410
Comorbidities, %				
HCV	0.88 (0.84-0.91)	<0.0001	0.81 (0.77-0.85)	<0.0001
HBV	0.99 (0.89-1.11)	0.8900		
Complication				
Aspiration Pneumonia	20.6 (19.7-21.6)	<0.0001	14.30 (13.63-15.01)	<0.0001
Septic Shock	24.7 (23.1-26.5)	<0.0001	9.15 (8.41-9.97)	<0.0001
Rhabdomyolysis	6.7 (6.4-7.1)	<0.0001	3.22 (3.04-3.42)	<0.0001
Anoxic Brain Injury	69.1 (63.1-75.7)	<0.0001	15.5 (13.70-17.50)	<0.0001
Income, %				
First quartile	Ref			
Second quartile	1.04 (1.00-1.09)	0.0710		
Third quartile	1.00 (0.95-1.06)	0.9170		
Fourth quartile	0.89 (0.83-0.95)	<0.0001		
Insurance, %				
Government	Ref			
Private	0.97 (0.93-1.02)	0.2940		
Self- Pay	1.15 (1.06-1.25)	0.0010		
Others	0.93 (0.86-1.02)	0.1220		
Region, %				
North East	Ref			
Mid-West/North Central	1.43 (1.28-1.60)	<0.0001	1.57 (1.42-1.74)	<0.0001
South	1.70 (1.55-1.86)	<0.0001	1.62 (1.49-1.75)	<0.0001
West	1.67 (1.53-1.82)	<0.0001	1.38 (1.27-1.50)	<0.0001
Hospital Teaching Status, %				
Rural	Ref			
Urban Non-Teaching	0.96 (0.87-1.05)	0.3910		
Urban Teaching	0.99 (0.90-1.09)	0.8450		
Length of Stay, Days	1.03 (1.04-1.05)		1.04 (1.03-1.04)	<0.0001
≥Mortality, %	44.80 (41.78-48.06)		20.17 (18.34-22.18)	<0.0001

Cost and mortality of opioid overdose on IMV during hospitalization

The hospitalization costs associated with IMV use were significantly higher than those without IMV (28,117.81±373.53 vs 8412.97±122.32) (p<0.001). Opioid overdose patients requiring IMV also had a higher length of stay by 8.9 ± 0.1 days as compared to those who were not on IMV 5.1 ± 0.0 days even after adjusting for other variables (p<0.001) (Table 2). Also, a higher in-hospital mortality rate (13.4% vs 0.3%) can be seen in those on IMV (p<0.001) and it remained so after adjusting for other factors (OR 20.17 (18.34-22.18) (p<0.001) (Table 2).

## Discussion

The increase in the prevalence of opioid overdose patients by 31% and in those on IMV by 38% during the study period, 2010-2015, is astronomically high. This also applies to the mortality rate, which is also on the increase. The number of opioid overdose deaths in 2018 is reported to be about six times the rate in 1999 [10]. Even though there was a slight decrease in death by 2% from 2017-2018, this trend has continued to rise year after year.

Beginning with the baseline demographics, this study reports that white males and ages 40-64 years were the most hospitalized patients, and this is consistent with other similar studies where they have been predominantly white, male, older age groups admitted for opioid overdose [11-13]. However, an ICU study reported having more white females in their fifth decade [3]. Apart from age and gender, other risk factors have been identified as independent risk factors to consider. A study conducted among New York hospitals reported that white race and low level of poverty contributed to adverse fatal opioid overdose [14]. Our study also supports these findings where opioid overdose patients were more among those in the lowest quartile of income (p< 0.0001). Although after adjusting for other factors, this statistical significance was lost.

Much of the emphasis on the prevention of opioid overdose has been on the need to equip bystanders and emergency workers, such as emergency medical technician staff and the police, on the need to give naloxone on the field when a person is suspected to have overdosed [15-16]. However, the critical care needs of hospitalized patients with opioid overdose is beginning to gain more traction especially in areas where the cases of overdose victims are still very high [7]. This study clearly addresses the need to provide interventions that support more critical care resources and the expansion of primary prevention measures. The use of mechanical ventilation is said to be higher among opioid overdose patients as compared to other forms of drug overdose [17]. Two smaller sample size ICU studies with 178 and 42 opioid overdose patients reported that 85% and 88% of admissions in the ICU required IMV, respectively [3,18]. On the other hand, our large study reported that 162,345 out of about 2,528,751 opioid overdose patients on hospitalization required IMV intervention. The disparity in numbers might be because our study compared rates with all hospitalized patients with opioid overdose rather than just those in the ICU.

Another thing to consider, especially when IMV is needed in hospitalized patients, is the cost of hospital care. As was earlier stated, the overall cost of caring for opioid overdose patients in the ICU increased from an average of $58,000 in 2009 to $92,400 in 2015, which is about 58% more in spending per patient [7]. A study that was the first to be done in New York City to describe ICU resource utilization in patients with acute drug overdose averaged the cost of care for patients with acute drug intoxication to be about $16,080 per patient higher than many other studies compared [17]. This differs, however, from the average cost of our hospitalized patient, which is about $9, 674, and similar to other studies [3,17]. However, in our patients who had IMV, the cost of hospital care was much higher ($28, 117 v $8,413) (p< 0.0001). This further emphasizes the need for better strategies to limit spending and hospital costs, especially in potential IMV users. Examples of such measures will include the screening of high-risk comorbidities and the screening of those with baseline demographic predictors early during hospitalization. This will ensure that clinicians provide early airway management and keep a close watch on such patients to prevent complications during hospital care of opioid overdose patients.

The most seen complications among hospitalized patients were assessed in our study. Our findings were stunning. Even after adjusting for other predictors, these complications were associated with increased odds of IMV use. In our study, the most common complication was aspiration pneumonia (3.7%) followed by rhabdomyolysis (2.9%) and then septic shock and anoxic brain injury (1.2% and 0.8% respectively). A study similarly found these complications among opioid overdose patients admitted in the ICU. About a quarter experienced aspiration pneumonia while 15% had rhabdomyolysis, 8% had an anoxic brain injury and 6% had septic shock [7]. If these complications are not taken care of early, they may lead to an increase in the mortality rate.

The causes of death in an ICU study by Pfister showed that brain injury accounted for more than half of mortality cases, acute respiratory failure was responsible for about a quarter, and the other causes of death were myocardial infarction and sepsis [3]. The mortality rate of those who were on IMV (13.4%) compares relatively to those in a related ICU study put at 10.1% [3]. However, the overall mortality rates in our study and in the no IMV group were relatively low (1.2% and 0.3%, respectively) as compared to those who were on IMV (13.4%). Another factor that was considered during analysis was the hospital length of stay, which was an average of 8.9 days (p< 0.0001) in those who were on IMV. This is similar to another study of heroin overdose patients in the ICU with a mean hospital length of stay reported as eight days [18].

Limitations identified include the means of data collection of these patients, which was through the hospital billing system ICD-9 CM. This method is prone to missing some admissions for opioid-related complications. Also, being administrative data, this creates the opportunity to miss out on some clinical details that might be useful when analyzing what forms of intervention can be proffered. Such clinical details would be things like how many times was the patient on IMV in the same admission. This might give us more information about how the patient was managed and what complications they experienced during hospitalization. However, one of the strengths of our study is the availability of both clinical and demographic data across the United States and the fact that it is a large database. This large database increases the power of the study and the generalizability of our research findings. This study answers some of the questions asked in previous studies about predictors of hospitalizations in opioid overdose patients, which will guide policy and interventions that will limit hospital admissions and avoid a prolonged length of stay in the health care facility [3].

## Conclusions

The prevalence of opioid overdose and the need for IMV increased over the five-year study period reflecting an increase in the relatively high in-hospital mortality of opioid overdose patients on IMV. These findings emphasize the need for a better understanding of the predictors of IMV use in hospitalized opioid patients to create a more strategic approach for their hospital care. There is a need to provide interventions that support more critical care resources and the expansion of primary prevention measures.
